# A First Assessment of *Mycobacterium tuberculosis* Genetic Diversity and Drug-Resistance Patterns in Twelve Caribbean Territories

**DOI:** 10.1155/2014/718496

**Published:** 2014-03-10

**Authors:** Julie Millet, Shirematee Baboolal, Elisabeth Streit, Patrick E. Akpaka, Nalin Rastogi

**Affiliations:** ^1^WHO Supranational TB Reference Laboratory, Unité de la Tuberculose et des Mycobactéries, Institut Pasteur de Guadeloupe, 97183 Abymes, Guadeloupe, France; ^2^Department of Para-Clinical Sciences, Faculty of Medical Sciences, The University of the West Indies, Saint Augustine, Trinidad And Tobago

## Abstract

With the exception of some French-speaking islands, data on tuberculosis (TB) in the Caribbean are scarce. In this study, we report a first assessment of genetic diversity of a convenience sample of *Mycobacterium tuberculosis* strains received from twelve Caribbean territories by spoligotyping and describe their drug-resistance patterns. Of the 480 isolates, 40 (8.3%) isolates showed resistance to at least one anti-TB drug. The proportion of drug-resistant strains was significantly higher in The Bahamas (21.4%; *P* = 0.02), and Guyana (27.5%; *P* < 0.0001), while it was significantly lower in Jamaica (2.4%; *P* = 0.03) than in other countries of the present study. Regarding genetic diversity, 104 distinct spoligotype patterns were observed: 49 corresponded to clustered strains (2 to 93 strains per cluster), while 55 remained unclustered among which 16 patterns were not reported previously. Combining the study results with regional data retrieved from the international SITVIT2 database underlined a connection between frequency of certain *M. tuberculosis* phylogenetic lineages and the language spoken, suggesting historical (colonial) and ongoing links (trade, tourism, and migratory flows) with European countries with which they shared a common past.

## 1. Introduction

Key factors in the control of tuberculosis (TB) are rapid detection, adequate therapy, and contact tracing to arrest further transmission, as well as active drug-resistance surveillance to avoid emergence of multidrug and extensively drug-resistant TB [1]. In developed countries where tuberculosis is on the decline, many molecular epidemiological studies were performed to strengthen TB prevention and control measures [2]. Most of these studies are based on a combination of traditional and molecular epidemiological studies to identify predominant* Mycobacterium tuberculosis* complex clones causing disease in different subpopulations and locations. Although TB molecular epidemiological studies and description of TB bacilli genetic diversity have been conducted in the French-speaking Caribbean permitting to obtain a good insight into its origin and spread [3], studies on genetic diversity of* M. tuberculosis* strains and TB epidemiology remain scarce in the rest of the Caribbean. Considering that such studies might help provide evidence-based data crucial for the development of policies and strategies for effective TB prevention and control, the main objective of this study was therefore to make a first assessment of* M. tuberculosis* genetic diversity using spoligotyping and to determine drug-resistance patterns in twelve Caribbean territories.

## 2. Materials and Methods

### 2.1. Bacterial Isolates and Drug-Susceptibility Testing

The study is based on a convenience sample of 480* Mycobacterium tuberculosis* complex (MTBC) strains (one isolate per patient) from the following Caribbean territories: Bahamas (*n* = 28), Belize (*n* = 6), Barbados (*n* = 14), Dominica (*n* = 1), Guyana (*n* = 91), Jamaica (*n* = 85), St. Lucia (*n* = 10), St. Kitts (*n* = 1), Suriname (*n* = 103), Trinidad and Tobago (*n* = 132), Turks and Caicos Island (*n* = 3), and St. Vincent and the Grenadines (*n* = 6). Sputum and clinical specimens from Belize, Dominica, Jamaica, St. Kitts, Nevis, St. Lucia, St. Vincent and the Grenadines, Trinidad and Tobago, and Turks and Caicos Islands were referred to the Mycobacteriology Laboratory at the Caribbean Epidemiology Centre (CAREC) for culture, identification, and drug susceptibility testing (DST), while culture on LJ slants from the Bahamas, Barbados, Suriname, and Guyana was addressed to CAREC for identification and DST.* M. tuberculosis* strain collection was constituted over a 24-month period (September 2006–August 2008) or isolated from patients in Trinidad and Tobago over a 12-month period (October 2006–September 2007).* Mycobacterium tuberculosis *complex (MTBC) was identified on the basis of its selective inhibition by p-nitro-a-acetylamino-*β*-hydroxypropiophenone (NAP) [4] in the Bactec 460TB system (Becton Dickinson, Franklin Lakes, USA). Drug susceptibility testing (DST) to first-line drugs was performed using the Bactec 460TB system at the following concentrations: streptomycin (SM), 2.0 *μ*g/mL; isoniazid (INH), 0.1 *μ*g/mL; rifampicin (RIF), 2.0 *μ*g/mL; ethambutol (EMB), 2.5 *μ*g/mL. Although this collection of MTBC strains may be considered a convenience sample, it should be underlined that repeat cultures as well as specimens without basic demographic data were not included.

### 2.2. Molecular Typing and Database Comparison

Spoligotyping was carried out to study the polymorphism of the Direct Repeat (DR) locus as previously described [5] on bacterial DNA samples shipped to the Pasteur Institute of Guadeloupe. The patterns obtained were compared by using the SITVIT2 proprietary database of the Pasteur Institute of Guadeloupe, which is an updated in-house version of the publicly released SpolDB4 [6] and SITVITWEB [7] databases. In this database, Spoligotype International Type (SIT) designates an identical pattern shared by two or more patient isolates, whereas “orphan” designates patterns reported for a single isolate that does not correspond to any of the strains recorded in the database repository.

### 2.3. Phylogenetical Analysis

Major phylogenetic clades were assigned according to signatures provided in SpolDB4 [6], slightly revised by the addition of 5 “new rules” for definition of variants within 62 existing lineages/sublineages in SITVITWEB [7]. These include specific signatures for various* M. tuberculosis* complex members, as well as rules defining major lineages/sublineages for* M. tuberculosis stricto sensu*, that is, the Beijing clade, the Central Asian (CAS) clade and two sublineages, the East African-Indian (EAI) clade and nine sublineages, the Haarlem (H) clade and three sublineages, the Latin American-Mediterranean (LAM) clade and 12 sublineages, the “Manu” family and three sublineages, the S clade, the IS*6110*-low-banding X clade and four sublineages, and an ill-defined T clade with five sublineages. MTBC population structure was studied by drawing a map to underline the distribution of major lineages (T, EAI, LAM, X, Haarlem, and Beijing) within our settings as well as by comparing the results obtained with regional data retrieved from the international SITVIT2 database (*n* = 2653 strains from 6 surrounding territories with the following distribution: Cuba *n* = 256, Haiti *n* = 404, Guadeloupe *n* = 342, Martinique *n* = 158, Venezuela *n* = 927, and French Guiana *n* = 566). The BioNumerics software Version 6.6 (Applied Maths NV, Sint-Martens-Latem, Belgium) was used to build the minimum spanning trees (MST) based on spoligotyping data. MST is an undirected network in which all of the isolates are linked together with the fewest possible linkages between nearest neighbors.

### 2.4. Statistical Analysis

Statistical analyses were performed using STATA version 10.1. Associations between variables were assessed using Chi-square analysis and Fisher's exact test. *P* values ≤0.05 were considered statistically significant.

## 3. Results

### 3.1. Demographical and Epidemiological Data

Information on gender was available for 460 (95.8%) isolates with a male to female sex ratio of 346/114 or 3.03. As shown in Table 1, there was a wide range of sex ratios among the countries surveyed; they varied from 1.0 in Belize to 4.6 in Suriname.

Age was available for 450 (93.8%) of the isolates and these ranged from 4 months to 83 years with a mean age of 39.9 years. Mean ages were significantly not equal from one country to another and ranged from 23.6 years in Belize to 45.2 years in St. Vincent and the Grenadines (*P* = 0.02). The distribution of cases among the various age groups was nonhomogenous and was higher for the age groups 15–34 and 35–54 years which comprised 37.8% (*n* = 170/450) and 43.1% (*n* = 194/450) of all the isolates, respectively. The proportion of patients in the age group 15–34 years was significantly higher in Jamaica than in the other countries of the study (49.4% versus 35.3%, resp.; *P* < 0.02), while the proportion of patients in the age group 35–54 years was significantly higher in The Bahamas and Guyana than in other countries of the study (64.3%, *P* < 0.02 and 53.9%, *P* < 0.05, resp.).

Results of HIV serology were available for 317 (66.0%) of the isolates, of which 86 (27.1%) were positive. Based on available HIV results in TB patients in the various countries, the TB/HIV coinfection was relatively high when compared to developed countries and showed rates as high as 44.4% were shown in Guyana followed by 42.9% in The Bahamas, 30.6% in Trinidad and Tobago, 21.4% in Suriname, 16.9% in Jamaica, and 14.3% in Barbados. The proportion of TB/HIV coinfected patients was significantly higher in The Bahamas (42.9%; *P* = 0.05), while this rate was significantly lower in Jamaica (16.9%; *P* = 0.05) as compared to other countries studied. Data on HIV status for the isolates from Belize, St. Lucia and St. Vincent and the Grenadines, Turks and Caicos, Dominica, and Saint Kitts and Nevis were not available.

### 3.2. Drug Resistance

Of the 480 isolates, 40 (8.3%) isolates showed resistance to at least one anti-TB drug (Table 1). The majority of these drug resistant strains were from Guyana (25/40; 62.5%) followed by strains from The Bahamas (6/40; 15.0%) and Belize (4/40; 10.0%). The proportion of drug-resistant strains was significantly higher in The Bahamas (21.4%; *P* = 0.02), and Guyana (27.5%; *P* < 0.0001), while it was significantly lower in Jamaica (2.4%; *P* = 0.03) than in other countries of the present study. Note that the high proportion of drug-resistant strains reported in Table 1 for Belize (66.7%;*P* = 0.001) should be considered with caution due to the small number of isolates studied (*n* = 6), and should be reconfirmed on larger datasets.

### 3.3. Spoligotyping Based Clustering of Isolates

Spoligotyping of the 480 isolates of the study generated 104 distinct patterns (individual spoligotype patterns and phylogenetical lineages observed are detailed in Supplemental Table S1; see Supplementary Material available online at http://dx.doi.org/10.1155/2014/718496). A total of 425 isolates or 88.5% of the typed isolates were grouped in 49 clusters (2 to 93 isolates per cluster). Among the remaining 55 unclustered isolates, 39 or 8.1% belonged to preexisting SIT designations in the database, while 16 or 3.3% were not yet reported in the SITVIT2 database and represented orphan profiles. Overall, the proportion of clustered isolates was relatively high (88.5%; *n* = 425/480) and ranged from 33.3% in St. Vincent and the Grenadines to 90.2% in Trinidad and Tobago (Table 1); differences were statistically significant among the countries with significantly lower values, for example, in St. Vincent and the Grenadines (33.3% versus 89.0%; *P* = 0.002), The Bahamas (64.3% versus 88.1%; *P* = 0.002), Suriname (79.6% versus 87.8%; *P* = 0.03), and Jamaica (81.2% versus 88.9%; *P* = 0.05).

### 3.4. Major Shared-Types

Among the 49 clusters, 30 included 2 to 4 isolates each and these were defined as minor spoligotypes (Supplemental Table S1). Major spoligotypes were defined as SITs that were shared by 5 or more isolates each and there were 19 such spoligopatterns (Table 2). The geographic distribution of major SITs was as follows.

- The most predominant clone SIT53, belonging to the ill-defined T lineage, was found in 9 of the 12 countries and was the major lineage in Barbados (28.6%; *n* = 4/14), Jamaica (16.5%; *n* = 14/85), Guyana (49.5%; *n* = 45/91), and St. Lucia (50.0%; *n* = 5/10).
- The second most common clone SIT566 (*n* = 74) belonging to an undefined (unknown) lineage was seen exclusively in Trinidad and Tobago and made up 56.1% of the strains isolated in this country.
- The third most prevalent clone SIT131 (*n* = 25) also belonging to the ill-defined T family was seen in isolates from 3 Caribbean countries, Guyana (*n* = 13), Suriname (*n* = 11), and Barbados (*n* = 1). It should be noted that the isolate from Barbados was a Guyanese national currently residing in Barbados; therefore, SIT131 in this study was restricted to only Guyana and Suriname.
- The fourth major pattern SIT1340 belonged to the EAI lineage and was seen exclusively in isolates from Guyana (*n* = 6/91 or 6.6%) and Suriname (*n* = 18/103 or 17.5%). Isolates of the Beijing lineage (SIT1, *n* = 17) were observed in 5 countries: Trinidad and Tobago (*n* = 6), Suriname (*n* = 5), Jamaica (*n* = 4), and The Bahamas and Guyana (*n* = 1 each).
- The Beijing strain originating from Guyana was a multidrug-resistant (MDR) strain, while one of the strains originating from Jamaica was from an Asian national, and the strain originating from The Bahamas was from a Tanzanian national living in The Bahamas (results not shown).
- Lastly, we can underline that SIT2934 (*n* = 8), SIT2550 (*n* = 7), and SIT2935 (*n* = 5) were only found in Trinidad and Tobago and that SIT2016 (*n* = 11), and SIT2406 (*n* = 9) were isolated only from patients living in Jamaica.

### 3.5. Phylogenetic Lineages

Lineage determination was performed following published rules [6, 7]. Of the 480 isolates, 395 (82.3%) could be classified among one of the lineage present in the database (Supplemental Table S1). A total of 17.7% of the isolates (*n* = 85/480), mainly from Trinidad and Tobago (SIT566 and SIT450, *n* = 75/85 or 88.2%), were labeled as “unknown” with an undefined lineage attribution. The remaining unlabeled 10 isolates corresponded to SIT237 (*n* = 1, Suriname), SIT450 (*n* = 1, Turks and Caicos), and SIT1084 (*n* = 1, Suriname), as well as 7 orphan profiles. Major lineages observed in the Caribbean were as follows: T (*n* = 160/480 or 33.3%), EAI (*n* = 90/480 or 18.8%), X lineage (*n* = 36/480 or 7.5%), LAM (*n* = 27/480 or 5.6%), Haarlem (*n* = 27/480 or 5.6%), LAM10-CAM (*n* = 23/480 or 4.3%), and Beijing (*n* = 17/480 or 3.5%). Other minor lineages were also present in our setting such as the AFRI lineage (*n* = 2/480), BOV (*n* = 5/480), CAS (*n* = 4/480), Manu (*n* = 2/480), and the S lineage (*n* = 2/480).

### 3.6. Geographic Distribution

**Figure 1:**
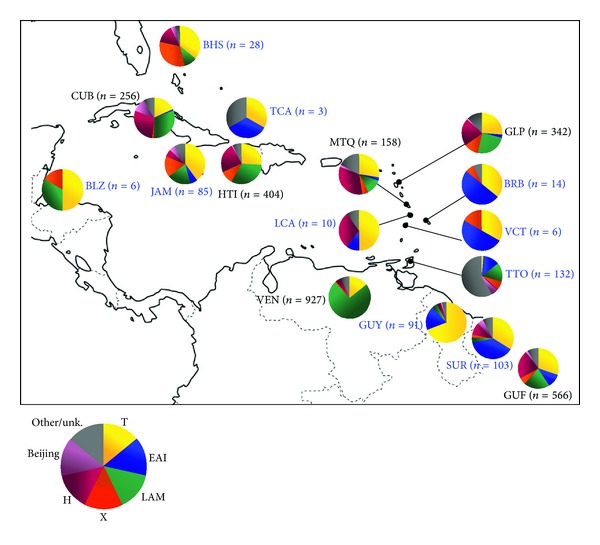
Geographical distribution of genotypic lineages in 10 out of 12 countries of the study (with a total number of isolates >1) and 6 surrounding territories in the SITVIT2 database (Cuba *n* = 256; French Guiana *n* = 566; Guadeloupe *n* = 342; Haiti *n* = 404; Martinique *n* = 158; Venezuela *n* = 927). Countries with a blue color font correspond to those included in the present study.

We also studied the distribution of major lineages in our study versus results available in the SITVIT2 database for surrounding territories. Data on distribution of major lineages (T, EAI, LAM, X, Haarlem, and Beijing) for 10 out of the 12 Caribbean countries of our study as well as 6 surrounding territories were extracted from the SITVIT2 database (Figure 1).

- Strains of the T lineage were found in all the 16/16 territories studied and represented from 69.2% (Guyana) to 1.5% of the strains (Trinidad and Tobago), while the other lineages were differentially represented among territories.
- Strains of the EAI lineage predominated in Barbados (*n* = 7/14 or 50.0% of the strains), Suriname (*n* = 39/103 or 37.9% of the strains), and Saint Vincent (*n* = 3/6 or 50.0% of the strains), while this lineage represented less than 10% of the strains in Belize (*n* = 0/6), Cuba (*n* = 3/256 or 1.2% of the strains), Guadeloupe (*n* = 9/342 or 2.6% of the strains), Haiti (*n* = 0/404), Jamaica (*n* = 5/85 or 5.9% of the strains), Martinique (*n* = 4/158 or 2.5% of the strains), and Venezuela (*n* = 0/927).
- Strains of the LAM lineage predominated in Cuba (*n* = 79/256 or 30.9% of the strains), Haiti (*n* = 131/404 or 32.4% of the strains), and Venezuela (*n* = 683/927 or 73.8% of the strains), while strains of the Haarlem lineage only predominated in Martinique (*n* = 53/158 or 33.5% of the strains).
- Interestingly, territories with more than 10% of their strains classified among the EAI lineage were localized in the South of the Caribbean arc and in northern South America, in an area ranging from Saint Lucia to French Guyana.
- On the other hand, a predominance of the T lineage strains was observed in territories localized in the area ranging from Guadeloupe to the Bahamas: *n* = 90/342 or 26.3% of the strains in Guadeloupe, *n* = 34/85 or 40.0% in Jamaica, and *n* = 10/28 or 35.7% in the Bahamas; and of LAM lineage strains in Haiti (*n* = 131/404 or 32.4%) and Cuba (*n* = 79/256 or 30.9%).

### 3.7. Association of Clades and Languages

**Figure 2:**
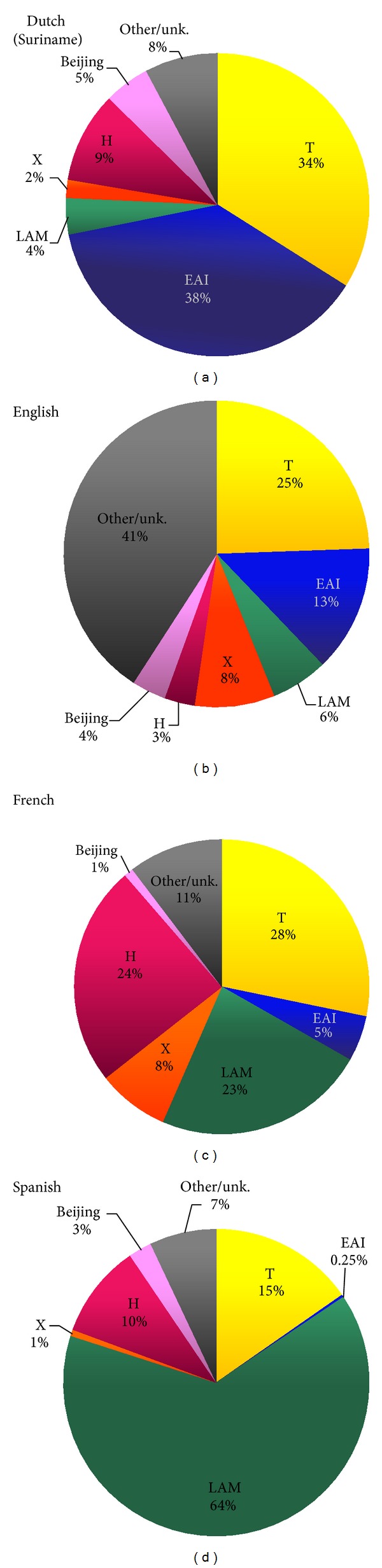
Distribution of genotypic lineages by official language of the isolation country: Dutch (Suriname); English (The Bahamas, Barbados, Belize, Guyana, Jamaica, St. Vincent and the Grenadines, Trinidad and Tobago, Turks and Caicos Islands); French (French Guiana, Guadeloupe, Haiti, Martinique and St. Lucia); Spanish (Cuba, Venezuela).

Significant differences were observed in relation to the prevalence of the major spoligotype families and the official language spoken in the studied areas (Figure 2).

- LAM lineage strains were isolated in high proportions in Spanish-speaking territories (*n* = 762/1183 or 64.4% of the isolates) versus Dutch (Surinam *n* = 4/103 or 3.9%), English (*n* = 30/499 or 6%), and French-speaking territories (*n* = 345/1480 or 23.3%); the difference being statistically significant (*P* < 0.001).
- The T family on the other hand was overrepresented in Suriname (*n* = 35/103 or 34%) versus Spanish-speaking territories (*n* = 181/1183 or 15.3%; *P* < 0.05). In French- and English-speaking countries, T family strains represented 28.2% (*n* = 418/1480; *P* = 0.2*-NS*) and 24.4% (*n* = 122/499; *P* = 0.05) of the cases, respectively.
- The Haarlem clade was mainly associated with French-speaking areas where it accounted for 24.3% of the isolates as compared to 9.9% (*n* = 117/1183; *P* < 0.001) in Spanish-, 9.7% (*n* = 10/103; *P* < 0.001) in Dutch-, and 3.2% (*n* = 16/499; *P* < 0.001) in English-speaking areas.
- The EAI family made up the majority of the strains isolated in Suriname (37.9% or *n* = 39/103) but was significantly less frequent in English- (*n* = 67/499 or 13.4%; *P* < 0.001) and French- (*n* = 74/1480 or 5%; *P* < 0.001) speaking countries and very rare in Spanish-speaking areas (*n* = 3/1183 or 0.25%; *P* < 0.001).
- The prevalence of X clade isolates ranged from 8.4% (*n* = 42/499) and 7.8% (*n* = 116/1480), respectively, in English- and French-speaking areas to 1.9% (*n* = 2/103) in Dutch-speaking and 0.68% (*n* = 8/1183) in Spanish-speaking areas.
- Lastly, It should be noted that owing to the high prevalence of a specific pattern SIT566 (*n* = 74) in Trinidad and Tobago of unknown lineage designation [8, 9], statistics for the English-speaking countries should be taken with caution.

### 3.8. Evolutionary Relationship among Spoligotypes and Lineages

**Figure 3:**
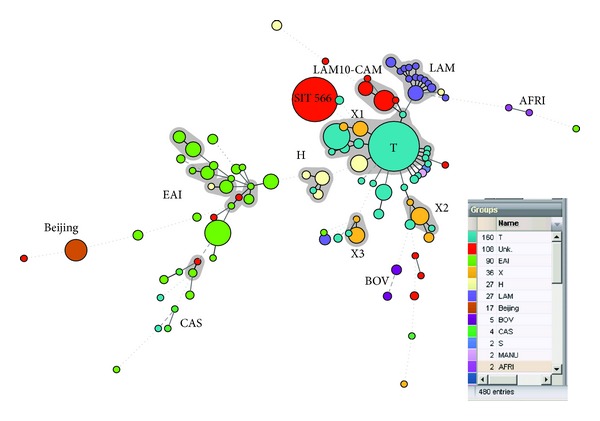
A Minimum spanning tree (MST) showing evolutionary relationships among spoligotypes and lineages observed in our setting (*n* = 480 strains).

We drew a minimum spanning tree (MST) based on spoligotypes obtained in our studys (Figure 3). We observed major genogroups in the MST corresponding to the following lineages: T, X1, X2, X3, LAM, LAM10-CAM, H, EAI, AFRI, CAS, and Beijing and minor groups containing Bovis, S, Manu, and AFRI. Not considering the SIT566 strains of unknown lineage [8, 9], two major genogroups belonging to T (*n* = 160) and LAM (*n* = 27) lineage strains are in central positions in the MST with tight links between patterns (1 change between strains of the LAM group and 1 or 2 changes between strains of the T group) and shared a fair proportion of the strains in the Caribbean (*n* = 157/480 or 32.7% of the strains). On the other hand, strains belonging to another major genogroup EAI (*n* = 90 strains) were highly scattered suggestive either of its stepwise introduction in the Caribbean over time or of its local evolution in endemic areas following early introduction.

Last but not least, one can also notice on the MST that (i) the LAM10-CAM group was external to the LAM group, (ii) X1, X2, and X3 were unrelated groups, and (iii) Beijing, EAI, AFRI, CAS, and Bovis groups were in peripheral positions.

## 4. Discussion

In this study both conventional and molecular tools were used to determine the characteristics of TB isolates and infection in a number of Caribbean countries. Although the sample size for many of the small islands taken individually remains small, the global number of isolates studied (*n* = 480) constitutes a reasonable sample to make a first assessment of* M. tuberculosis *genetic diversity and drug-resistance patterns in the Caribbean. The results obtained show that the study population was characterized by a rather high male to female sex ratio—a trend that has already been reported but in a lesser proportion in a study conducted over 14 countries [10]. Although partly due to the high values observed in Suriname and Trinidad and Tobago, the higher proportion of males among the TB population of patients in our study could also be due to the precarious lifestyles of many of the males as reported elsewhere in association with male sex [11–13].

When compared to the extremely high TB incidence in some hotspots in the Caribbean such as Haiti (incidence of 306/100,000) and Dominican Republic (incidence of 69/100,000), the TB incidence in most of the studied countries remains stable at a moderate level [1]. Nonetheless, the high TB/HIV coinfection rate observed globally (nearly one-third of the study population or 27.1%) was alarming. High proportions of TB/HIV coinfected patients were observed in the present study (from 13.5% to 44.4%), which is in agreement with prevailing HIV/AIDS pandemic in this area. Indeed, the Caribbean region has been categorized as having the second highest incidence of HIV infection in the world after sub-Saharan Africa, and HIV prevalence among adults is estimated to be 1% [14]. In view of these findings, it is imperative to establish and maintain a close collaboration of the anti-TB and anti-HIV programs in these countries. The WHO has recommended intensified interventions to address TB and HIV in countries where coinfection rates exceed 5%, and voluntary HIV testing of all TB patients is recommended where the coinfection rate exceeds this number [1, 15, 16]. In the Caribbean most of the countries have implemented this strategy and are well on their way to achieving testing of all tuberculosis patients for HIV infection [17].

Additionally, HIV patients with symptoms of tuberculosis should be routinely screened for TB. Nonetheless, a significant proportion of HIV patients may screen negative for TB using smear microscopy alone [18]; hence a culture is therefore needed to confirm the presence of infection. In this context, the absence of culture facilities in many of the Caribbean countries is therefore a major limitation for the confirmation of TB in all HIV patients. Despite the moderate-to-low incidences of TB observed in the Caribbean, these were higher than that of 6/100,000 observed in the French Caribbean islands of Guadeloupe and Martinique [3]. Since the proportion of TB/HIV coinfected patients is reportedly on rise (this information is available for 8 countries out of 12 Caribbean territories studied between 2005 and 2007), utmost attention is needed to prevent TB associated with HIV/AIDS in the Caribbean [15, 16].

Despite the global expansion in coverage of drug-resistance surveillance, data on drug resistance was still unavailable for more than 100 countries throughout the world in 2009, including most of the Caribbean countries [19]. Nonetheless, the overall level of drug resistance observed in our study (8.3%) was lower than the worldwide average (11.1%) in the global study [19], as well as the rate of 12.9% reported for the two French islands, Guadeloupe and Martinique [3]. Although drug resistance has been reported from several countries in the Caribbean, data reported to WHO on drug resistance during the study period is often lacking; for example, a single case of MDR-TB from Trinidad and Tobago was reported in 2006 [20]. In the present study, resistance to 1st-line antituberculosis drugs was seen in 6 countries, five of which had more than 5 cases of TB/100,000 population (Table 1). The highest level of any drug resistance was seen in Guyana (*n* = 25/91 or 27.5% cases), with a very high proportion of MDR-TB strains since 19/25 drug-resistant strains showed combined resistance to isoniazid and rifampin; thus Guyana accounted for 86.4% of all MDR-TB strains observed in our study (*n* = 19/22).

However, despite the higher proportion of drug-resistant TB cases, Guyana showed similar rates of clustering when compared with other countries of this study, suggesting that these cases probably corresponded to acquired drug resistance in unlinked patients. This finding is in agreement with a previous study that reported resistance to at least one anti-TB drug in 22.2% of the tested Guyanese MTBC isolates and MDR-TB in 11.1% of the strains [21]. The authors attributed these high levels of resistance to inadequate monitoring and followup of patient treatment and to poor management of the TB control program. In contrast, no drug-resistant strains were found in Trinidad and Tobago and only one monoresistant isolate was obtained in Suriname in the present study despite the high rate of TB-HIV coinfection in both countries. Nonetheless considering that our study is based on a convenience sample, it might be too speculative to make any definitive comments.

Spoligotyping was used to explore the genetic diversity of* M. tuberculosis* clinical isolates in our study. Although it may correctly identify “outbreak” episodes as well as reflect the spread of the disease due to human migratory movements, it is known to overestimate clustering of isolates [2]. In the present investigation, the global clustering rate attained 88.5% which is rather high, suggesting the need for second-line typing methods such as MIRU-VNTRs [22] in order to identify the exact rate of ongoing transmission in the Caribbean. Nonetheless, our aim in this first assessment in the 12 Caribbean territories was not TB molecular epidemiology, but rather limited to genetic diversity. The results obtained (Table 2 and Supplemental Table S1) showed that the T, EAI, and X lineages were the major clades observed. Regarding the ill-defined T family, apart SIT53/T1 sublineage known to be a well-recognized ubiquitous pattern in SpolDB4 and SITVITWEB databases [6, 7] and present in 9/12 countries in our study, we also identified a geographically localized clone corresponding to SIT131/T1 (Table 2). It was exclusively present in Guyana (*n* = 13) and Suriname (*n* = 11), since the only SIT131 strain found in Barbados was also isolated from a Guyanese national residing in Barbados. A search in the SITVIT2 database showed that beyond Guyana and Suriname, this SIT is commonly isolated in patients in French Guyana, which shares a common frontier with Suriname highlighting transborder circulation of this clone.

Another interesting observation in our study was the predominance of EAI6-BGD1 sublineage among EAI strains since this lineage is known to predominate in Bangladesh [23]. Isolates belonging to this clade were seen mainly in Suriname and Guyana countries with large communities of East Indians among their population (37.0% and 43.5% of East Indians, resp.; https://www.cia.gov/library/publications/the-world-factbook/index.html). Interestingly, although EAI strains were seen in Trinidad and Tobago, this specific sublineage was absent and lineages such as EAI1-SOM and EAI2-Manilla were seen. This difference in EAI sublineages (clearly visible by its highly scattered grouping in the MST drawn in Figure 3) seen in the Caribbean islands with important proportion of communities of East Indian descent probably highlights that people from different regions of the Indian subcontinent were brought to different islands/countries during indentureship.

Distribution of genotypic lineages by official language of the isolation country (Figure 2) showed that both English- and French-speaking territories had equal proportion of X lineage strains. Known to have phylogeographical specificity for patients of Anglo-Saxon descent [3], this lineage has been present in Guadeloupe for decades and likely is a relic or the discontinuous periods of English colonization between 1691 and 1816 (http://www.axl.cefan.ulaval.ca/amsudant/guadeloupe.htm) Interestingly, all the 3 known sublineages (X1, X2, and X3 shown as scattered genogroups in the MST drawn in Figure 3) were present in the Caribbean: X1 was mainly found in Guadeloupe, Haiti, Martinique, Trinidad and Tobago, Jamaica, Suriname, and St. Vincent and the Grenadines; X2 was only observed in Jamaica and Trinidad and Tobago (and absent in the French-speaking Caribbean), while X3 was present in the French-speaking Caribbean as well as in The Bahamas, Trinidad and Tobago, and St. Vincent and the Grenadines.

The Beijing lineage was first described in 1995 [24], and is notorious as a cause of major outbreaks worldwide, often involving drug-resistant variants [25, 26]. However, the prevalence of Beijing family strains was reported to be less than 1% in many of the South American countries [27], with the exception of Peru (as high as 9.3% of all strains) due to its historical relationship with East Asia [28]. Despite this known scarcity of the Beijing lineage strains in South America, we observed Beijing genotype strains in 5 countries, accounting for 17/480 or 3.5% of the isolates (Table 2). Contrary to previous reports [26, 29], Beijing strains found in the Caribbean were not associated with an elevated level of drug resistance—indeed 16/17 strains were pansusceptible and only a single isolate corresponded to a case of MDR-TB. This low proportion of Beijing strains in our study is consistent with published data for the French departments: a study covering the period from 1994 to 2003 found that Beijing lineage strains were absent in Guadeloupe and Martinique, and only 1.6% of the isolates from French Guiana belonged to this lineage [3]. Nevertheless, there might be a rise in the Beijing lineage strains over the recent years due to increased migratory flows from Eastern Asia since a more recent study (2004-2005) reported 6.3% Beijing isolates from Martinique and 2.2% from French Guiana [30]. Knowing the propensity of Beijing strains to develop drug-resistance in absence of optimal therapy and management of patients [26, 29], the detection of Beijing strains in the Caribbean in 3.5% of the TB cases should be taken with utmost caution.

In our opinion, the variation in proportions of strains of different phylogenetic lineages from one territory to another could reflect specificities of their historical past in conjunction with the insular nature of the island nations, a hypothesis supported by the observed association of clades and languages (Figure 2). Indeed, the official language in a given Caribbean country is determined by its colonial past which indirectly explains why the LAM family, particularly common in the Mediterranean Basin and strongly associated to Spanish descent [3, 6], accounts for more than half of the isolates obtained in Spanish-speaking areas, followed by French speaking areas (owing to Latin and Mediterranean heritage), but comparatively scarce in English- and Dutch-speaking settings. On the opposite, the high prevalence of EAI strains in Suriname and the English-speaking territories most likely is a consequence of the large-scale immigration of Asian workers after the abolition of slavery in these countries. We have already commented on the specificities of the X lineage above, but there might be a point to comment further on the separation of LAM versus LAM10-CAM in the MST shown in Figure 3. Despite its name, this particular lineage is no longer considered part of the LAM family and was recently reclassified as the Cameroon lineage [31] and is essentially limited to Jamaica and Trinidad and Tobago within Caribbean. Another specific example of exclusive nature of certain endemic MTBC clones is the predominance of SIT566 (belonging to a yet undefined lineage); it was highly specific for Trinidad and Tobago since all the 74 such strains were isolated in this country (Table 2). Interestingly, only other 11 SIT566 strains found so far in the international SITVIT2 database were all traced back to TB patients in USA; origin of patients was known in 6 cases and all were from Trinidad and Tobago confirming the phylogeographical endemicity of SIT566 in a single Caribbean country so far [8, 9].

In conclusion, while historic migratory flows have left their traces in present MTBC clade distribution in the Caribbean, ongoing migratory movements may be important factors to be taken into account when designing anti-TB programs. This is particularly true for countries receiving important numbers of immigrants from areas with elevated levels of drug-resistant TB like Guyana or from high burden countries such as Haiti. Haitians are the most important group of inner Caribbean migrants nowadays (http://atlas-caraibe.certic.unicaen.fr/fr/page-250.html) and originating from one of the world's TB hotspots not only they have an elevated chance of developing TB at some point in their life, but also in disease propagation since high-incidence hotspots are known to play an important role in propagating TB epidemics [32].

## Supplementary Material

Supplemental Table S1: Spoligotype patterns and phylogenetical lineages observed among 480 M. tuberculosis clinical isolates isolated in 12 countries of the Caribbean. A total of 425/480 or 88.5% isolates were grouped in 49 clusters (2 to 93 isolates per cluster), while 55/480 or 11.5% were unclustered. Furthermore, 395/480 or 82.3% isolates were correctly classified among one of the lineages present in the database.

## Figures and Tables

**Table 1:** Epidemiological, demographic, and genotyping results corresponding to *M. tuberculosis* isolates (*n* = 480) from 12 Caribbean territories.

Characteristic	Total sample	TTO^a^	SUR^a^	GUY^a^	JAM^a^	BHS^a^	BRB^a^	LCA^a^	VCT^a^	BLZ^a^	TCA^a^	DMA^a^	KNA^a^
Number of isolates	480	132	103	91	85	28	14	10	6	6	3	1	1
Gender													
Male	346	105	83	58	59	18	8	7	4	2	1	1	0
Female	114	26	18	22	24	10	5	3	1	3	1	0	1
Sex ratio (M/F)	3.0	4.0	4.6	2.6	2.5	1.8	1.6	2.3	4.0	0.7	1.0	NA^b^	NA^b^
Age													
≤14 yrs (%)	8 (1.8%)	0	2 (2.0%)	1 (1.3%)	3 (3.8%)	0	0	1 (10.0%)	0	1 (20.0%)	0	0	0
15–34 yrs (%)	170 (37.8%)	40 (31.7%)	38 (37.6%)	29 (37.2%)	39 (49.4%)	9 (32.1%)	7 (50.0%)	2 (20.0%)	1 (16.7%)	3 (60.0%)	1 (100.0%)	0	1 (100.0%)
35–54 yrs (%)	194 (43.1%)	55 (43.7%)	41 (40.6%)	42 (53.8%)	24 (30.4%)	18 (64.3%)	4 (28.6%)	4 (40.0%)	4 (66.7%)	1 (20.0%)	0	1 (100.0%)	0
55–74 yrs (%)	71 (15.8%)	30 (23.8%)	16 (15.8%)	6 (7.7%)	13 (16.5%)	0	2 (14.3%)	3 (30.0%)	1 (16.7%)	0	0	0	0
>75 yrs (%)	0	1 (0.8%)	4 (4.0%)	0	0	1 (3.6%)	1 (7.1%)	0	0	0	0	0	0
Mean age (yrs)	39.9	42.9	41	37.5	36.9	40.3	38.1	44	45.2	23.6	NA^b^	NA^b^	NA^b^
Number of isolates	450	126	101	78	79	28	14	10	6	5	1	1	1
HIV serology													
HIV positive^c^	86/317	37/121	18/84	8/18	10/59	12/28	1/7	NA^b^	NA^b^	NA^b^	NA^b^	NA^b^	NA^b^
% of TB/HIV coinfection	27.1%	30.6%	21.4%	44.4%	16.9%	42.9%	14.3%	NA^b^	NA^b^	NA^b^	NA^b^	NA^b^	NA^b^
DST													
Any resistance	40/480	0/132	1/103	25/91	2/85	6/28	2/14	0/10	0/6	4/6	0/3	0/1	0/1
MDR	22	0	0	19	0	0	1	0	0	2	0	0	0
Genotyping													
% of clustered isolates	88.5%	90.2%	79.6%	87.9%	81.2%	64.3%	71.4%	80.0%	33.3%	66.7%	0.0%	NA^b^	NA^b^
Major SIT^d^	SIT53	SIT566	SIT1340	SIT53	SIT53	SIT53, SIT70	SIT53	SIT53	NC	NC	NC	NC	NC
Major clade^d^	T	Unk.	EAI	T	T	T, X3	EAI	T	NC	NC	NC	NC	NC
New SITs^e^	13	2	5	0	4	1	0	0	1	0	0	0	0
Orphan^f^	16	1	5	1	6	1	0	2	0	0	0	0	0
Unknown^g^	88	75	6	1	5	0	0	0	0	0	1	0	0
Beijing^h^	17	6	5	1	4	1	0	0	0	0	0	0	0

^a^Distribution of the 480 strains of the study among the 12 Caribbean countries and territories. The three-letter codes for Trinidad and Tobago (TTO, *n* = 132), Suriname (SUR, *n* = 103), Guyana (GUY, *n* = 91), Jamaica (JAM, *n* = 85), The Bahamas (BHS, *n* = 28), Barbados (BRB, *n* = 14), Saint Lucia (LCA, *n* = 10), Saint Vincent and the Grenadines (VCT, *n* = 6), Belize (BEL, *n* = 6), Turcs and Caicos islands (TCA, *n* = 3), Dominica (DMA, *n* = 1), and Saint Kitts and Nevis (KNA, *n* = 1)are according to http://en.wikipedia.org/wiki/ISO_3166-1_alpha-3.

^b^Not Available (NA).

^c^Proportion of patients with HIV positive status among those with known HIV status.

^d^Major SITs and clades sharing the most important number of strains; not commented (NC) from territories with less than 10 isolates.

^e^Proportion of newly created SITs after comparing spoligotypes with SITVIT2.

^f^Proportion of orphan profiles after comparing spoligotypes with SITIVIT2.

^g^Proportion of profiles with no clade attribution according to predefined rules.

^h^Proportion of strains with a spoligotyping profile corresponding to the Beijing lineage.

**Table 2:** Description of predominant shared types (*n* = 19) representing clustered *M. tuberculosis* clinical isolates (5 or more strains) and their distribution among the 12 countries of the study.

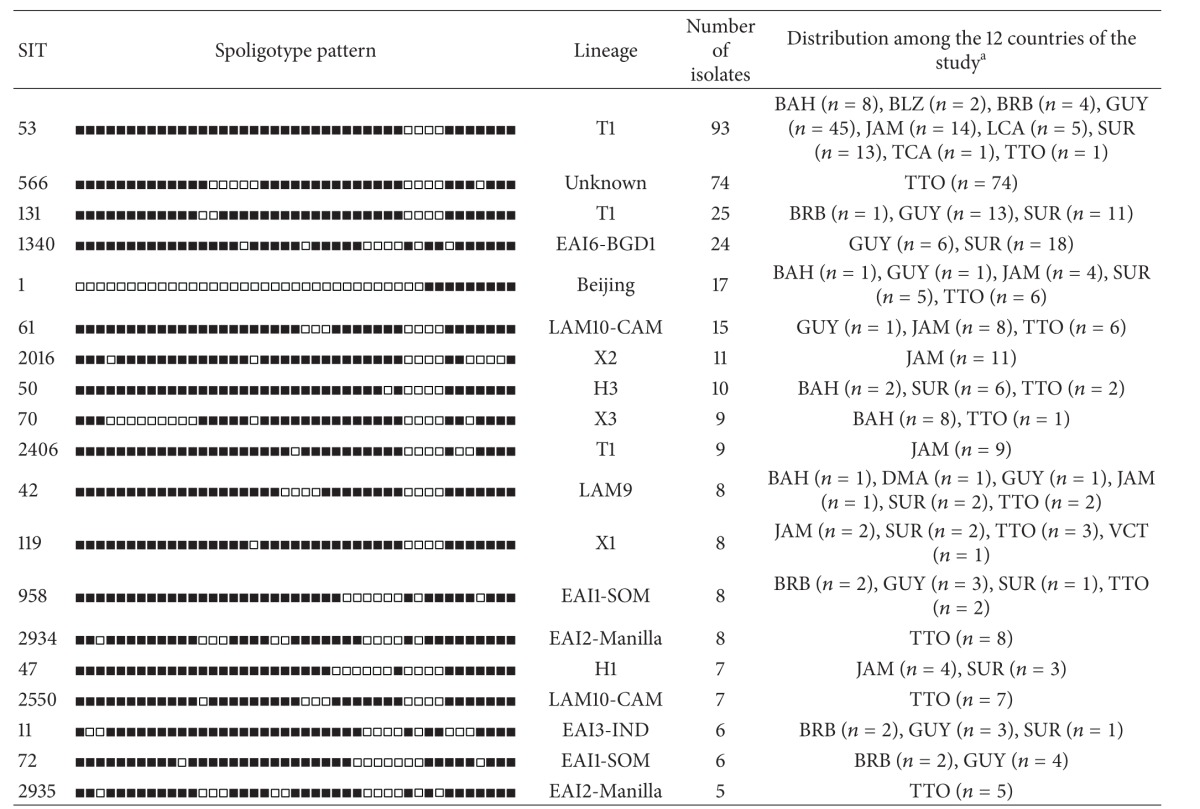

^a^The 3-letter country codes are according to http://en.wikipedia.org/wiki/ISO_3166-1_alpha-3; note that the 19 predominant SITs contain 350/480 strains of the study.
