# Supplementary material for: A First Assessment of Mycobacterium tuberculosis Genetic Diversity and Drug-Resistance Patterns in Twelve Caribbean Territories
Source: Biomed Res Int. 2014 Mar 10;2014:718496. doi: 10.1155/2014/718496 (PMC3985416; doi:10.1155/2014/718496)
Supplement: Supplementary file 1 — Supplemental Table S1: Spoligotype patterns and phylogenetical lineages observed among 480 M. tuberculosis clinical isolates isolated in 12 countries of the Caribbean. A total of 425/480 or 88.5% isolates were grouped in 49 clusters (2 to 93 isolates per cluster), while 55/480 or 11.5% were unclustered. Furthermore, 395/480 or 82.3% isolates were correctly classified among one of the lineages present in the database. [file 718496.f1.pdf]

Supplemental Table S1: Spoligotype patterns and phylogenetical lineages observed among 480 *M. tuberculosis* clinical isolates isolated in 12 countries of the Caribbean.

Lineage (Total, %)	Sublineage	SIT	Binary Spoligotype pattern	Number of strains
T (n=160, 33.3%)				
	T1	53	■■■■■■■■■■■■■■■■■■■■■■■■■■■■■■■■□□□□■■■■■■■	93
	T1	131	■■■■■■■■■■■■□□■■■■■■■■■■■■■■■■■■□□□□■■■■■■■	25
	T1	2406	■■■■■■■■■■■■■■■■■■■■■□■■■■■■■■■■□□□□■□□■■■■	9
	T1	51	■■■■■■■■■■■■■■■■■■■■■■■■■■■■■■■■□□□□■■■□□□□	3
	T1	2982	■■■■■■■■■■■■■■■■■■■■■□■■■■■■■■■□□□□□□□□■■■■	3
	T1	519	■■■■■■■■■■■■■■■■■■■■■■■■■■■■■■■□□□□□□■■■■■■	2
	T1	1526	■■■□□□□□□□□□□■■■■■□□■■■■■■■■■■■■□□□□■■■■■■■	2
	T1	7	□■■■■■■■■■■■■■■■■■■■■■■■■■■■■■■■□□□□■■■■■■■	1
	T1	77	■■■■□□□■■■■■■■■■■■■■■■■■■■■■■■■■□□□□■■■■■■■	1
	T1	205	■■■□■■■■■■■■■■■■■■■■■■■■■■■■■■■■□□□□■■■■■■■	1
	T1	453	■■■■■■■■■■■■■■■■■■■■□■■■■■■■□■■■□□□□■■■■■■■	1
	T1	516	■■■■■■■■■■■■■■■■■■■■■■■■■■■□■■■■□□□□■■■■■■■	1
	T1	521	■■■■■■■■■■■■■■■■■■■■■■■■■■■■■■■■□□□□■■□□□■■	1
	T1	Orphan	■■■■■■■■■■□■■■■■■■■■■■■■■■□□□□□□■□□□□■■■■■■■	1
	T1	Orphan	■■■■■■■□□■■■■■■■■■■■■■■■■■■■□□■■□□□□■■■■■■■	1
	T1	Orphan	■■■□□□□■■■■■■■■□□■□■□■□□□□□□□□□□□□■■■■■■■■■	1
	T2	736	■■■■■■■■■■■■■■■■■■■□■■■■■■■■■■■■□□□□■■■□■■■	1
	T2	2322	■■■■■■■■■■■■■■■■■■■■■■■□□□□□⑦■■■□□□□■■■□■■■	1
	T3	37	■■■■■■■■■■■■□■■■■■■■■■■■■■■■■■■■□□□□■■■■■■■	3
	T5	44	■■■■■■■■■■■■■■■■■■■■■■□■■■■■■■■■■□□□□■■■■■■■	1
	T	73	■■■■■■■■■■■■□■■■■■■■■■■■■■■■■■■■□□□□■■■□■■■	3
	T	2983	□□■■□■■■■■■■■□■■■□■■■■■■■■■■■■■■□□□□■■■□■■■	2
	T	197	■■■□□□□□□□□□□■■■■□■■■■■■■■■■■■■■□□□□■■■□■■■	1
	T	1081	■■■■■■■■■■■■□■■■■□■■■■■■■■■■■■■■□□□□■■■□■■■	1
	T	2296	■■■□■■■■■■■■■■■■■■■■■■■■■■■■■■■■□□□□■■□□□□■	1
EAI (n=90, 18.8%)				
	EAI1-SOM	958	■■■■■■■■■■■■■■■■■■■■■■■■■■□□□□□□■□■■■■■□■■■	8
	EAI1-SOM	72	■■■■■■■■■■□■■■■■■■■■■■■■■■■□□□□□□□■■■■■□■■■	6
	EAI1-SOM	349	■■■■■■■■■■■■□■■■■■■■■■■■■■■■■□□□□■□■■■■■□■■■	3
	EAI1-SOM	48	■■■■■■■■■■■■■■■■■■■■■■■■■■■■⑦⑦⑦□■□■■■■■□■■■	1
	EAI1-SOM	2986	■■□■■■■■■■■■■■■■■■■■■■■■□□■■□□□□■□■■■■■□■■■	1
	EAI2-Manilla	2934	■■□■■■■■■■■■□□□■■■■□□■■■■■■■□□□□■□■■■■■■■■■	8
	EAI2-Manilla	2935	■■□■■■■■■■■■□□□■■■■□□■■■■■■■□□□□■□■□■■■■■■■	5
	EAI2-Manilla	19	■■□■■■■■■■■■■■■■■■■□□■■■■■■■□□□□■□■■■■■■■■■	2
	EAI2-nonthaburi	89	■■□■■■■□□□□□□□□□□□□□□□□□□■■■□□□□■□■■■■■■■■■	3
	EAI3-IND	11	■□□■■■■■■■■■■■■■■■■■■■■■■■■■□□□□■□■■□□□■■■■	6
	EAI3-IND	2990	■□□■■■■■■■■■■■□□□□■■■■■■■■■■□□□□■□■■□□□■■■■	2
	EAI3-IND	414	■□□■■■■■■■■■■■■■■■■■■■■□■■■■□□□□■□■■□□□■■■■	1
	EAI5	138	■■■■■■■■■■■■■■■■■■■■■■■■■■■■□□□□■□■■■■■□□□□	3
	EAI5	763	■■■■■■■■■■■■□□□□□□■■■■■■■■■■□□□□■□■■■■■□□□□	3
	EAI5	2987	■■■■■■■■■■■■■■■■□□□□□□□□■■■■□□□□■□■■■■■■■■■	2
	EAI5	126	■□□■■■■■■■■■■■■■■■■■■■■■■■■■□□□□■□■■■■■■■■■	1
	EAI5	Orphan	■■■■■■□□□□□□□□□□■■■■□□□□□□□□□□□□□■■■□□□■■■■	1
	EAI5	Orphan	■■■□□□□□□□□□■■■■■□■■■□□■■■■■■■■■□□□□■■■□□□□	1
	EAI5	Orphan	■■■□□□□■■■■■■□□□□□□□□□□□□□□□□□□□□□□■■■□□□□	1
	EAI6-BGD1	1340	■■■■■■■■■■■■■■■■□■■■■■□■■■■■□□□□■□■■□■■■■■■	24
	EAI6-BGD1	2985	■■■■■■■■■■■■■■■■■■■■■■□□□□□■□□□□■□■■□■■■■■■	2
	EAI6-BGD1	1409	■■■■■■■■■■■■■■■■■■■■■■□■■■■■□□□□■□■■□■□■■■■	1
	EAI6-BGD1	1970	■■■■■■■■■■■■■■■■■□■■■■□■■■■■□□□□■□■■■■■■■■■	1
	EAI7-BGD2	2991	■■□■■■■■■■■■■■■■■■■■■■□■□□□■□□□□□□■■□■■■■■■	2
	EAI	2989	■■■■■■■■■□■■■■■■■■■■■□□□□□□□□□□□□□■■□■■■■■■	2
Haarlem (n=27, 5.6%)				
	H1	47	■■■■■■■■■■■■■■■■■■■■■■■■■□□□□□□■□□□□■■■■■■■	7
	H1	45	■■■■■■■■■■■■■■■■■■■■■■■□■□□□□□□■□□□□■■■■■■■	3
	H1	62	■■■■■■■■■■■■■■■■■■■■■■■■■□□□□□□■□□□□■■■□■■■	2
	H3	50	■■■■■■■■■■■■■■■■■■■■■■■■■■■■■■□■□□□□■■■■■■■	10
	H3	800	■■■■■■■□□□□□□□□□□□□■■■■■□□□□□□□■□□□□■■■■■■■	3
	H3	Orphan	□■■■■■■■■■□■■■■■■■■■■■■■■■■□□□□□□□■■■■■□■■■	1
	H3	Orphan	■■■■■■■■■■■■■■■■■■■■■□□□■■□□■■■■□□□□■■■■■■■	1
LAM (n=27, 5.6%)				
	LAM1	20	■■□■■■■■■■■■■■■■■■■■□□□□■■■■■■■■□□□□■■■■■■■	1
	LAM1	2506	■■□■■■■■■■■■■□■■■■■■□□□□■■■■■■■■□□□□■■■■■■■	1
	LAM2	2199	■■□□■■■■■■■■□■■■■■■■□□□□■■■■■■■■□□□□■■■■■■■	3
	LAM2	17	■■□■■■■■■■■■□■■■■■■■□□□□■■■■■■■■□□□□■■■■■■■	1
	LAM3	950	■■■□□□□□□□□□□■■■■■■■□□□□■■■■■■■■□□□□■■■■■■■	4
	LAM3	376	□■■■■■■■□□□■■■■■■■■■□□□□■■■■■■■■□□□□■■■■■■■	1
	LAM4	60	■■■■■■■■■■■■■■■■■■■■□□□□■■■■■■■■□□□□■■■□■■■	1
	LAM5	93	■■■■■■■■■■■■□■■■■■■■□□□□■■■■■■■■□□□□■■■■■■■	1
	LAM6	64	■■■■■■■■■■■■■■■■■■■■□□□□■■■■□■■■□□□□■■■■■■■	1

	LAM6	176	■■■■■■■■■■■■□■■■■■■■■□□□□■■■■□■■■□□□□■■■■■■■	1
	LAM9	42	■■■■■■■■■■■■■■■■■■■■□□□□■■■■■■■■□□□□■■■■■■■	8
	LAM9	509	■■■■■■■■■■■■■■■■■■■■□□□□■□■■■■■■□□□□■■■■■■■	2
	LAM9	162	■■■■■■■■■■■■■■■■■■■■□□□□■■■■■■■■□□□□■■□■■■■	1
	LAM	1710	■■■■■■■■■■■■□■■■■■■■■□□□□■■■■■■■■□□□□■■■□■■■	1
Cameroon (n=23, 4.8%)				
	LAM10-CAM	61	■■■■■■■■■■■■■■■■■■■■■■□□□■■■■■■■□□□□■■■■■■■	15
	LAM10-CAM	2550	■■■■■■■■■■■■□■■■■■■■■■■■□□□■■■■■■■□□□□■■■■■■■	7
	LAM10-CAM	2981	■■■■■■■■□□□□□□□□□■■■■■□□□■■■■■■■□□□□■■■■■■■	1
X (n=36, 7.5%)				
	X1	119	■■■■■■■■■■■■■■■■■□■■■■■■■■■■■■■■□□□□■■■■■■■	8
	X1	1823	■■■■■■■■■■■■■■■■■□■■■■■■■■■■■■■■□□□□■■■■■■□	2
	X2	2016	■■■□■■■■■■■■■■■■■□■■■■■■■■■■■■■■□□□□■■□□□□■	11
	X2	478	■■□□□■■■■■■■■■■■■□■■■■■■■■■■■■■■□□□□■■□□□□■	3
	X2	137	■■■■■■■■■■■■■■■■■□■■■■■■■■■■■■■■□□□□■■□□□□■	1
	X3	70	■■■□□□□□□□□□■■■■■□■■■■■■■■■■■■■■□□□□■■□■■■■	9
	X3	92	■■■□□□□□□□□□■■■■■□■■■■■■■■■■■■■■□□□□■■■■■■■	1
	X	199	■■■□□□□□□□□□■■■■■□■■■■■□□□□□□□□□□□□□□□□□□■■	1
Beijing (n=17, 3.5%)				
	Beijing	1	□□□□□□□□□□□□□□□□□□□□□□□□□□□□□□□□□□■■■■■■■■■	17
Other/Unk (n=100, 20.8%) *				
	Other/Unk	–	–	100

* Note that the number n=100 for the “other/unknown” group corresponds to the sum of patterns with no clade attribution (see Table 1), as well as profiles other than the 7 majors lineages described in the text (note that 16 orphan patterns shown in Table 1 are equally split in this supplemental table as 8 being classified among various lineages while the other 8 belong to the “other/unknown” group).
